# Complete Republication: Recent Updates to CSE Recommendations for Promoting Integrity in Scientific Journal Publications: 7 Ways to Integrate Diversity, Equity, and Inclusion Into Scholarly Publishing

**DOI:** 10.5888/pcd20.230051

**Published:** 2023-03-23

**Authors:** Leonard Jack

**Affiliations:** 1 Office of Medicine and Science, National Center for Chronic Disease Prevention and Health Promotion, Centers for Disease Control and Prevention, Atlanta, Georgia

This article is a republication of a peer-reviewed article featured in the Council of Science Editors’ journal, *Science Editor. *The purpose of the republication is to expand dissemination on best practices in diversity, equity, and inclusion in scholarly publishing. The original citation is as follows: Jack Jr L. Recent updates to CSE recommendations for promoting integrity in scientific journal publications: 7 ways to integrate diversity, equity, and inclusion into scholarly publishing. Sci Ed. 2022;45:117-120. https://doi.org/10.36591/SE-D-4504-02

*The Council of Science Editors’ (CSE) Recommendations for Promoting Integrity in Scientific Journal Publications was first published in 2006, and the full document was updated in 2009 and again in 2012. In 2018, the CSE Editorial Policy Committee (EPC) began making updates on a rolling basis as new sections were added or existing sections updated to reflect new information or best practices. This updated method for amending the document allows for more rapid dissemination of its contents so that recommendations can be quickly put into practice in journal operations. In this column, the reader is advised of a recent update that provides guidance on the importance of advancing best practices in scholarly publishing. Content in this update, while condensed, was taken largely verbatim from the CSE’s Recommendations for Promoting Integrity in Scientific Journal Publications. However, readers are encouraged to visit the full set of Recommendations for Promoting Integrity in Scientific Journal Publications:* https://www.councilscienceeditors.org/recommendations-for-promoting-integrity-in-scientific-journal-publications-.

## Introduction

The role of both intentional and unintentional bias in society, including in scientific publishing, is receiving increased attention and discussion.^1,2^ Content assessed for publication in scientific journals and articles eventually published are not immune from bias. In fact, bias against individuals because of their race, gender, religion, disability, education, institutional setting, career status, sexual orientation, spoken language, and other characteristics remains a pressing issue in scientific publishing.^3^ Emerging diversity, equity, and inclusion (DEI) best practices are becoming increasingly important to promote equitable actions that advance diversity of disciplines, racial and ethnic diversity, institutional diversity, interdisciplinary fields, gender diversity, geographic diversity, and linguistic and cultural diversity,^1^ as well as inclusion of perspectives represented by this diversity. This commentary provides a brief overview of new content in CSE’s Recommendations for Promoting Integrity in Scientific Journal Publications regarding DEI best practices in scholarly publishing (Figure).

**Figure:**
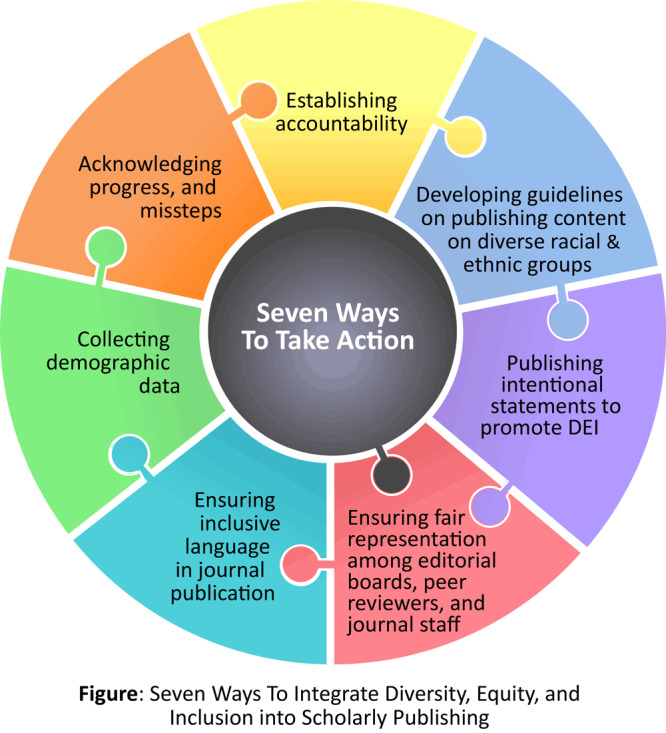
Seven ways to integrate diversity, equity, and inclusion into scholarly publishing.

## Operationalizing DEI

Journals can take steps toward achieving the important goals of DEI. In terms of ***diversity**,* journals should ensure diverse representation to provide feedback to the journal.^4^ Efforts should be made to go beyond familiar and often more comfortable representation to ensure diversity among staff leadership, external review panels, associate editors, editorial board members, statistics review committee members, guest editors, peer reviewers, subject matter consultants, and journal leadership and staff members. Journals should strive to achieve and maintain a commitment to advancing ***equity*** by proactively working to expand representation and thereafter listening and then implementing action steps in response to feedback particularly from diverse persons and/or those most affected by a practice, program, and/or policy, recognizing the contributions of all volunteers and staff, and providing a range of opportunities for others to lead and participate in key decision making.^4^ Being just and fair by seeking feedback from a range of diverse persons helps to create open dialogue among various partners both internal and external to the journal.^4^ Journals can ensure ***inclusion*** by taking proactive steps so that a range of individuals are and will continue to be part of discussions that identify a broad spectrum of ideas and perspectives.^4^ Encouraging such participation and engagement may help journals prevent any one paradigm, belief, or perspective in the science and practice of their field to dominate a journal’s decision making and, ultimately, the type of content it publishes.^4^

The following areas highlight some of the actions that can be taken to ensure DEI best practices and policies in scientific publishing:

- **Establish accountability:** Publishers, organizations, and journals must hold themselves accountable to become educated on effective ways to advance DEI best practices. Realistic DEI goals, objectives, and benchmarks to measure progress and opportunities for improvement should be established. Information collected to monitor progress should be transparent and used to provide updates to key individuals including the publisher (if applicable), the journal’s readership, and the public.
- **Develop DEI-related guidelines on conducting, reporting, and publishing scientific content on diverse racial and ethnic groups:** It is important that journals work extremely hard to ensure published content does no harm and does not convey disrespect. One way to avoid this harm is for journals to provide clear guidance to authors on reporting of race and ethnicity in medical and scientific publications.^3,5^ Guidance to authors should communicate that the reporting of race and ethnicity in published papers must not be provided in isolation. Rather, reporting race and ethnicity should be accompanied by the reporting of other less acknowledged and less reported factors that contribute to shaping health outcomes. These less reported factors include structural and social determinants of health (e.g., forms of racism, disparities, and inequities).
- **Publish intentional statement(s) to promote DEI in scientific publishing:** One key action a journal can take to demonstrate a public commitment to these practices is to publish a statement. This statement should delineate the areas around which the journal intends to advance DEI principles in its publication practices and operations. The statement is usually generated by the publisher, organization, and/or journal’s leadership. Such a publication memorializes a journal’s commitment to transparency and can be used to provide updates on progress and on challenges encountered.^6^
- **Ensuring fair representation among editorial boards, peer reviewers, authors, and journal staff:** There has been increased attention on who has a role in influencing or helping to determine which authors and articles are selected for publication.^4,7–10^ Journals have an important role in making improvements by increasing the diversity of membership in their editorial boards and among their associate editors, authors, and peer reviewers. Achieving these goals will require journals to actively work to identify and secure participation of individuals from diverse backgrounds and experiences who have the appropriate content expertise to serve in these capacities.
- **Ensuring inclusive language in journal publications: **There are several tools and resources that can assist journals in ensuring inclusive language is used in scientific publishing.^11–13^ This can assist journals with incorporating inclusive and nonbinary language as part of their publisher’s style guide and author guidance. Journals should become familiar with these resources and identify the most appropriate for use in publishing content in their journal. Using reliable resources on inclusive language is critically important for several reasons (see below).
- **Collecting demographic data:** Demographic data provide key metrics that make it possible to understand who is at the table helping to decide what is published, including journal leadership and staff and the individuals authoring submissions. Examples of demographic data that should be collected and reported include gender, age, race and ethnicity, education, geographic location, institution/affiliation, sexual identity, occupation, military status, disability status, and career status (early, mid-, and late career).^3^ Journal leadership and staff must recognize the possibility of data misuse. Therefore, there is a need to establish and maintain safeguards to protect sensitive data being collected.
- **Acknowledging progress, and missteps:** A journal’s readership may be the first audience to notice progress toward advancing DEI principles in a journal’s day-to-day operations, whether it be an increase in meaningful participation of diverse participants such as guest editors or on editorial boards, or the use of inclusive language in publications. Along the way to achieving such milestones, there are likely to be mistakes made that will serve as valuable lessons. And finally, journals may release publications that unintentionally contain insensitive content that is viewed as offensive, stereotypical, and harmful to the journal’s readership. To ensure transparency and build trust with their readership, authors, staff, and volunteers, journals should make it the norm to acknowledge not only progress achieved but also any missteps, including a sincere explanation of how and when missteps will be addressed and corrected.^4^

## Conclusion

In closing, this new DEI section of CSE’s Recommendations for Promoting Integrity in Scientific Journal Publications calls attention to resources available to assist journals at various stages of implementing DEI-centered activities. The following two DEI resources may be of use to journals:

**CSE Repository of Scholarly Resources on Diversity, Equity, and Inclusion: **CSE has generated a compilation of guidance resources, documents, and other materials providing information related to furthering diversity, equity, and inclusion in scholarly publishing in six categories^6^:

- DEI Committees of Trade/Professional Organizations in Scholarly Publishing
- DEI and Peer Review
- DEI Statements/Policies from Journals/Professional Associations/Publishers
- Bias, Discrimination, and Racism
- Data Collection on Diversity, Equity, and Inclusion
- Reporting Sex, Gender, and Race in Publications
- Inclusive Language Communication.

These resources are by no means exhaustive. Access to resources can be found at https://www.councilscienceeditors.org/dei-scholarly-resources.

**Coalition for Diversity and Inclusion in Scholarly Communication (C4DISC):** The C4DISC was founded by trade and professional associations that represent organizations and individuals working in scholarly communications.^13^ C4DISC was formed to discuss and address issues of diversity and inclusion within our industry. Their website is located at https://c4disc.org/.

There are major themes reflected in this initial DEI section that will likely continue to evolve: establishing accountability; developing DEI-related guidelines on reporting, and publishing scientific content on diverse racial and ethnic groups and other minoritized groups; publishing intentional statement(s) to promote DEI in scientific publishing; ensuring fair representation among editorial boards, peer reviewers, authors, and journal staff; ensuring inclusive language in journal publications; collecting demographic data among those who touch the publishing process (authors, journal staff, editors); and acknowledging progress and missteps. This new guidance was developed with the goal of providing journals with impactful ways to advance DEI in their day-to-day operations. This new guidance can be modified and expanded upon based on a journal’s unique direction, circumstances, and needs.

## Acknowledgement

I thank the members of the CSE Editorial Policy Committee for their assistance with these updates.
